# Role of Immunotherapy in the Treatment of Hepatocellular Carcinoma

**DOI:** 10.3390/curroncol32050264

**Published:** 2025-04-30

**Authors:** Irina Y. Dobrosotskaya, Rashmi Kumar, Timothy L. Frankel

**Affiliations:** 1 Section of Medical Oncology, Department of Medicine, Ann Arbor Veterans Healthcare System, Ann Arbor, MI 48105, USA; 2 Rogel Cancer Center, University of Michigan, Ann Arbor, MI 48109, USA; 3 Department of Surgery, University of Michigan, Ann Arbor, MI 48109, USA

**Keywords:** hepatocellular carcinoma, immunotherapy, tyrosine kinase inhibitor

## Abstract

Hepatocellular carcinoma is the most common primary liver tumor and is strongly related to underlying liver cirrhosis. Common etiologies include viral hepatitis, elevated alcohol consumption and metabolic diseases, all of which result in liver inflammation and scarring. Previously, systemic therapies for locally advanced or metastatic disease were limited to tyrosine kinase inhibitors with poor efficacy and rare cures. Recent advances have harnessed the power of the immune system to combat disease, resulting in improved outcomes and occasional cures. Here, we describe the recent clinical trials in immunotherapies for the treatment of hepatocellular carcinoma as first- and second-line therapies and in combination with other drug classes.

## 1. Introduction

In the human adult, the liver is an immunologic organ with primary functions in detoxification of metabolites, synthesis of clotting factors, and cholesterol homeostasis [1]. Uniquely, the liver is the only organ which receives blood from both the systemic and portal circulations. This aspect of liver physiology requires that the liver serve as a site of immunologic surveillance and simultaneous tolerance to a wide range of enteric pathogens and toxins. This unique immunologic landscape plays a significant role in the development and eventual microenvironment of liver cancers.

Hepatocellular carcinoma (HCC) is the most common primary liver tumor that occurs in the context of chronic liver inflammation as a result of alcoholic liver disease, metabolic dysfunction-associated hepatitis, hepatitis B virus, or hepatitis C virus. In essence, as >90% of HCC cases are attributed to chronic inflammation, underlying liver fibrosis is a fundamental driver of HCC [2]. Amongst solid tumors, HCC is known to have broad resistance against cytotoxic chemotherapy. Several studies have shown compensatory mechanisms unique to liver physiology that help HCC evade cancer cell death. These include and are not limited to increased genetic variability within HCC tumors, upregulation of drug efflux transporters, increased expression of DNA repair proteins to combat damage induced by chemotherapeutics, and alterations in drug metabolism through the cytochrome p450 enzymes [3].

Chronic inflammation is the predominant phenotype uniting known risk factors to the eventual development of HCC. The liver’s ability to self-regenerate along with chronic insult is thought to underly the eventual malignant transformation. Liver injury secondary to chronic inflammation also induces the fibrosis that characterizes cirrhosis. Prior studies have linked inflammatory cytokines including IL-6 and TNF alpha to hepatocyte signaling which may promote disease progression (reviewed in [4]). The source of these cytokines includes cytotoxic T lymphocytes (CTLs), a critical cell type in combating viral infections and one of the most common risk factors for HCC. The abundance of lymphocytes has prompted investigation into immune-based therapies for the treatment of HCC [5].

In addition to recognizing virally infected cells, CTLs are thought to be the primary effector mechanism for eradicating tumor cells. Malignant cells accumulate a series of genetic mutations which lead to the development of novel proteins expressed on the cell surface called neoantigens. These “non-self” proteins can be recognized by cognate T cell receptors on CTLs, triggering the release of destructive payload and cell lysis. Unfortunately, due to tumor intrinsic escape mechanisms, proteins on tumor cells or CTLs often begin to appear and block effective immune cell recognition. As part of an adaptive process to prevent overactivation of the immune system, these checkpoints silence effective immune surveillance and allow tumor growth and spread. The best studied of these are the checkpoints CTLA4 and PD1/PD-L1. Expressed constitutively on regulatory T cells but upregulated on activated CTLs, CTLA4 blocks the ability of activating CD28 from binding to CD80 and CD86, shutting down the immune reaction. A blocking antibody to CTLA4 can re-establish the activity of CD28 and was the first immune checkpoint inhibitor to receive approval from the Food and Drug Administration. Programmed death ligand 1 (PD-L1) binds to its cognate receptor programmed cell death protein 1 (PD1) to create a signaling axis that prevents activation of key immune mediators and can lead to CTL apoptosis. Monoclonal antibodies to block this interaction have led to CTL reactivation and effective tumor clearance with clinical efficacy in many solid tumor types. Here, we will review the recent trials in the treatment of HCC with a focus on immunotherapy.

## 2. Frontline Immune Checkpoint Inhibitor (ICI) Therapy in Advanced HCC: Single-Agent Regimens

We will first consider the evidence behind the use of ICI agents in the management of advanced, previously untreated HCC (Table 1). All these trials recruited previously systemically untreated patients with advanced HCC not amenable to curative or locoregional therapy. Only patients with good liver function, reflected in their Childs–Pugh Class A status, were eligible in these as well as the overwhelming majority of other HCC-directed ICI-based trials.

### 2.1. Nivolumab

CheckMate 040 was a multicenter, multi-national, open-label, multi-cohort phase 1/2 trial that recruited patients in Asia, Europe and North America [6]. The patients who were offered nivolumab were either sorafenib-naïve (n = 80) or pretreated with sorafenib (n = 154). They were offered nivolumab, with the dose in the expansion phase being 3 mg/kg every 2 weeks. Responses were seen in 20% patients, slightly higher in treatment-naïve than pretreated patients, and appeared to be numerically lowest (14%) in hepatitis B-infected individuals. Disease control was noted in 64% of patients, with numerically slightly better disease control in patients without viral hepatitis. Exploratory analysis showed that PD-L1 TPS of at least 1% was seen in 20%. Response rates were numerically slightly higher in these (26%) than non-expressors (19%). Most responses occurred within the first 3 months of therapy. A five-year follow-up [7] showed durability, with a median duration of response of 22.6 and 39.7 months in sorafenib-naïve and sorafenib-experienced patients, respectively, and respective median overall survival (OS) of 26.6 and 15.1 months. Interestingly, the survival curve plateaued at around 60 months. Treatment was also well tolerated, with serious treatment-related adverse event rates of 3–6%. These results established the clinical efficacy of nivolumab in HCC, in both frontline and sorafenib-pretreated settings.

#### CheckMate 459

The utility of single-agent nivolumab in the frontline setting was further investigated in a randomized, open-label CheckMate 459 trial, in which nivolumab administered at 240 mg every 2 weeks was compared to sorafenib [8]. This robust, well-balanced trial enrolled 743 patients and aimed at assessing overall survival as the primary endpoint. At the time of the initial publication, nivolumab- vs. sorafenib-treated patients had median OS of 16.4 months vs. 14.7 months, respectively, which did not reach statistical significance. However, the separation of the survival curves continued to widen over time, and longer follow-up may reach statistical significance, as supported by post hoc analysis. In total, 38 and 46% of nivo and sorafenib recipients moved on to the next line of therapy at time of progression, with 2% vs. 20% of the latter arm receiving immune-oncology agents. The utilization of checkpoint inhibitors in the second-line setting could have improved survival in the sorafenib arm. Progression-free survival (PFS) was twice as long with nivolumab vs. sorafenib, which was not statistically significant. Response rate was 15% in nivolumab-treated patients, higher than the 7% in sorafenib recipients. PD-L1 TPS was at least 1% in about 20% of patients, similar to the CheckMate 040 population. In contrast, response rates to nivolumab were more than twice in PD-L1 expressors over non-expressors (28 vs. 12%), both numerically higher than responses to sorafenib (9 and 7%). Serious treatment-related adverse events were seen in 12% of nivolumab-treated patients. The toxicity profile appeared slightly more favorable in the nivolumab group. Discontinuation due to treatment-related adverse events occurred in about 10% of patients in each arm. In summary, nivolumab showed clinical efficacy that appeared at least numerically superior to the then-standard frontline drug sorafenib, with comparable to improved safety. This study was not designed to establish non-inferiority, but it was implied by the data, albeit not statistically proven.

### 2.2. Pembrolizumab

#### Keynote-224 Cohort 2

This was one of the earlier trials that established the efficacy of pembrolizumab in advanced HCC in the frontline setting. This phase 2, open-label trial enrolled 51 patients from Europe and North America [9]. The patient characteristics were similar to other trials, with about one-third of patients bearing extrahepatic metastatic disease and about 40% with viral hepatitis. The RECIST-defined response rate of 23% was achieved, with median time to response of about 6 months. The responses were durable, with a median duration of response of 16 months, and half of the responders were still on therapy at the time of data cutoff (median follow-up of 27 months). Toxicity was in keeping with previously reported ICI effects.

### 2.3. Durvalumab

Himalaya was a global, open-label phase 3 trial with one of the treatment arms including single-agent durvalumab [10]. While the overall trial enrollment was 1171 patients, those receiving single-agent durvalumab numbered 389. This trial had a relatively higher proportion of patients with viral hepatitis (59%) and more patients with extrahepatic disease (53%) compared to other similar studies. The noninferiority of OS for durvalumab (16.56 mos) vs. sorafenib control (13.77 mos), a secondary endpoint, was met and sustained with longer follow-up [11], while superiority was not met despite a numeric edge over sorafenib. The objective response rate (ORR) was 17%, on par with the other ICIs discussed above. The median duration of response was 16.82 months, similar to that of pembrolizumab in cohort 2 of KEYNOTE-224. Time to response was faster than what was seen with other drugs, around 2 months. Similarly to other ICIs, the long duration of response in the responders was noted, with the upper limit being not reached. This was also reflected in the PFS which plateaued after about 18 months. The safety profile was consistent with existing data for durvalumab. Importantly, grade 3–4 treatment-related adverse events were much less frequent in the durvalumab arm as compared to sorafenib (12.9 vs. 36.9%), as were those leading to discontinuation (4.1 vs. 11%). There were no immune treatment-related adverse events (TRAEs) leading to death in the durvalumab arm.

### 2.4. Tislelizumab

RATIONALE-301 was yet another open-label, global phase 3 trial in systemically untreated advanced HCC patients, investigating the clinical utility of tislelizumab [12]. This anti-PD-1 monoclonal antibody was compared to sorafenib. This study enrolled a major proportion of patients with viral hepatitis, 76%, far exceeding this population in other similar trials. A majority of patients, around 60%, had extrahepatic disease. OS noninferiority was the primary endpoint, which was met. Similarly to the data on durvalumab above, the OS superiority endpoint was not met. The median OS in the tislelizumab arm was 15.9 vs. 14.1 mos. Interestingly, the survival curves started to separate after 12 months, and diverged progressively more with time, implying the long-term qualitative effect of the ICI. ORR was superior for tislelizumab at 14.3% vs. 5.4%, as was duration of response (DOR) at an impressive 36.1 vs. 11 months. Interestingly, median PFS was not significantly different, 2.1 vs. 3.4 months for tislelizumab and sorafenib, respectively. Tolerability and TRAE-related drug discontinuation was similar for tislelizumab as the other ICIs discussed above, at 6.2%. More patients received the next line of systemic therapy than other similar trials, 55–60%.

In conclusion, a number of ICIs have been proved to be effective in the management of HCC. Non-inferiority to the previous standard of care, sorafenib, especially in view of superior long-term survival in responders, makes these a valid therapeutic choice.

## 3. Frontline ICI Therapy in Advanced HCC: Combination Regimens

While the single-agent ICI regimens have activity in managing HCC, combination regimens offer superior efficacy albeit at the expense of higher toxicity (Table 2).

### 3.1. Atezolizumab Plus Bevacizumab

This was the first frontline combination adopted in clinical practice based on the results of the IMbrave150 trial [13]. This open-label, global phase 3 trial compared the efficacy of atezolizumab in combination with bevacizumab (n = 336) compared to then-standard frontline sorafenib (n = 165). The co-primary endpoints were OS and PFS. This trial enrolled a significant proportion of patients with macrovascular invasion, approximately 40% in each arm, with 26% of patients having varices at baseline, about half of which were treated at baseline. The initial report after median follow-up of 8.6 months showed statistically superior survival in the atezo-bev arm, with the hazard ratio for death being 0.58 (CI, 0.42–0.79), with a significantly improved OS rate at various time points. This outcome was confirmed after an additional 12 months of follow-up [14], with median OS of 19.2 vs. 13.4 months. PFS and ORR were also statistically superior in the atezo-bev arm. Remarkably, 8% of patients in the investigation arm achieved complete response at last follow-up (<1% in control arm). Interestingly, about 60% of the responses were registered in the first 3 months of follow-up, while almost 20% of patients achieved a response after week 24. This underscores the positive significance of stable disease in these patients. The longitudinal dynamic of responses is noteworthy. While about the same proportion of responders retained their response at 12 months (69 vs. 65%), this number rapidly tapered by 18 months in the sorafenib arm (22% vs. 51% in atezo-bev arm). Both initial and updated subgroup analyses showed that patients with viral hepatitis had superior OS, while those with other etiologies did not. Another noteworthy subgroup observation was that the degree of survival benefit conferred by atezo-bev was similar in patients with or without macrovascular invasion [14]. Significantly, overall survival in patients with PD-L1-negative status, defined as fewer than 1% of tumor cells expressing PD-L1, was not significantly improved in the experimental arm. Other subgroup comparisons did not reveal any significant contrasts. Physical functioning and role functioning were maintained remarkably longer in atezo-bev-treated patients. Both treatment regimens were similarly tolerable, with a dose density of 95 ± 7% for atezo, 93 ± 10% for bev, and 84 ± 20% for sorafenib, and treatment-related adverse events leading to discontinuation of either (15.5%) or both atezo and bev being 7% vs. 10.3% for sorafenib. All-causality grade 3–4 bleeding was seen in 6.4% of patients in the atezo-bev arm and 5.8% of the sorafenib arm. Treatment-related deaths were similar in both arms. Only about one-third of patients had their PD-L1 expression assessed, so this subgroup analysis was underpowered. Based on these results, atezo-bev is established as a standard of care of previously untreated HCC patients with good liver function (Childs–Pugh Class A, 5–6 points).

### 3.2. Tremelimumab Plus Durvalumab

As briefly described above, Himalaya was a global, open-label phase 3, three-arm trial comparing either tremelimumab-durvalumab combination (STRIDE regimen) or single-agent durvalumab with sorafenib as a control [10]. The efficacy of durvalumab was addressed above. The treme-durva arm enrolled 393 patients vs. 389 in the sorafenib arm. The proportion of patients with viral hepatitis was about 59%, that with macrovascular invasion was approximately 25%, and a significant proportion of patients had extrahepatic disease (53%). The primary endpoint of superiority of OS for the STRIDE regimen of durvalumab plus tremelimumab (16.4 mos) vs. sorafenib control (13.8 mos) was met and sustained with longer follow-up [11]. The subgroup analysis did not show superior efficacy in patients with underlying hepatitis C, contrary to IMbrave150. Durable responses, defined by survival more than 36 mos seen mostly in responders, were seen in 103 patients (26%), vs. 64 (16%) in the sorafenib arm. Time to response was faster than what was seen with other drugs, around 2 months. Similarly to other ICIs, the long duration of response in the responders was noted, with the upper limit being not reached. This was also reflected in the PFS which plateaued after about 18 months. Notably, subgroup analysis did not show much difference in PD-L1-positive and -negative patients for both STRIDE and durvalumab arms. Safety analysis showed that grade 3–4 treatment-related adverse events were less frequent in the treme-durva arm as compared to sorafenib (25.8 vs. 36.9%), as were those leading to discontinuation (8.2 vs. 11%). Treatment-related deaths occurred in 2.3% of treme-durva patients vs. 0.8% of sorafenib recipients and were caused mostly by autoimmune effects.

### 3.3. Nivolumab and Ipilimumab

The results of the first preplanned interim analysis of CheckMate 9DW, a phase 3, randomized, open-label trial comparing nivolumab–ipilimumab combination with sorafenib or lenvatinib in the frontline setting, were presented in an oral abstract form at the 2024 annual ASCO meeting [15]. The nivo-ipi combination produced superior OS of 23.7 vs. 20.6 mos, the survival gap widening with increasing follow-up time. The ORR of 36% was also superior to the 13% seen in the control arm, with median DOR also being significantly longer in the nivo-ipi arm (30.4 vs. 12.9 mos). Median PFS was around 9 months in each arm; however, the PFS rate was superior in the nivo-ipi arm, more so with longer follow-up. TRAEs leading to discontinuation occurred in 18 vs. 10% patients. Peer-reviewed publication of the final analysis is eagerly awaited. Of note, the FDA approved the nivo-ipi combination for frontline management of unresectable or metastatic HCC on 11 April 2025.

**Figure 1:**
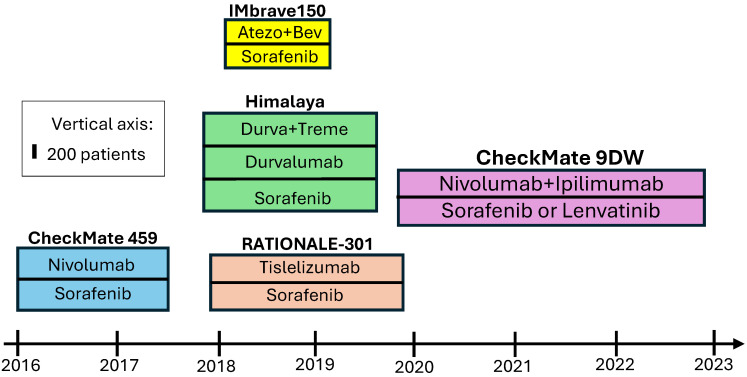
Timeline of phase 3 RCTs of frontline therapy including ICI vs. sorafenib/lenvatinib.

In summary, all three antibody combination regimens offer superior outcomes when compared to sorafenib and, in the case of CheckMate 9DW, lenvatinib. The efficacy also appears numerically superior to that of the single-agent ICIs described above, albeit at the cost of increased toxicity. The decision to offer a particular regimen, made as part of shared decision making with a patient, should consider the patient’s overall health and comorbidities. For instance, elevated risk of bleeding would make the atezolizumab-bevacizumab regimen less desirable than a non-VEGFR-altering regimen. Between-trial comparison is difficult, especially given relatively better outcomes in the control arm of the CheckMate 9DW trial, although this may be explained by the effectiveness of Lenvatinib, which was received by 85% of the control-arm patients. Pending a peer reviewed publication, nivo-ipi poses an attractive choice, given the superior survival outcomes as well as lack of bleeding risk in the absence of anti-VEGF activity. It has also been FDA-approved for this indication. While some oncologists will await the peer-reviewed publication of the data, this author (ID) considers nivo-ipi a good frontline option. It is important to also consider quality-of-life outcomes while making such decisions. Importantly, time to deterioration of global health status or quality of life was similar in the treme-durva and durva arms [10], emphasizing the utility of single-agent durvalumab in patients believed to be unfit for a combination therapy. The relative timeline of subject recruitment and numbers in these trials is reflected in Figure 1.

## 4. Frontline ICI Therapy in Advanced HCC: Immunotherapy/Tyrosine Kinase Inhibitor (TKI) Combinations

The idea of combining the immunomodulatory effect of ICIs with the TKI effects on cell signaling in the frontline setting was investigated with different ICIs and TKIs. As we will see, the addition of an ICI to TKIs does not improve outcomes in patients with HCC in the frontline setting. It did lead to an unfavorable toxicity profile.

### 4.1. Pembrolizumab and Lenvatinib

The combination of pembrolizumab and lenvatinib was compared to lenvatinib in LEAP-002, a randomized, double-blind phase 3 trial [16]. The dual primary endpoints were OS and PFS. After a median follow-up of 32 months, the OS outcomes of 21.2 and 19.0 mos for the investigational and control arms, respectively, did not meet the superiority threshold. The ORR was 26 vs. 17.5%, with a disease control rate of about 83% in each arm. The duration of response was 16.6 and 10.4 mos, respectively. The outcomes in the experimental arm resemble those from the pembrolizumab arm of Keynote-224 cohort 2 patients. Treatment-related grade 3–4 adverse events occurred in around 60% of patients in each arm, higher than in other combination regimens described above.

### 4.2. Atezolizumab and Cabozantinib

The combination of atezolizumab and cabozantinib was investigated in COSMIC-312 [17,18], an open-label, randomized, phase 3 trial of atezolizumab with cabozantinib compared to sorafenib. This combination also did not improve OS outcomes, with median OS of 15.4 and 15.5 mos in the two arms. PFS was improved in the atezo-cabo arm, at 6.8 vs. 4.2 mos. However, with other regimens offering a wealth of more effective options, this regimen is not a viable choice.

### 4.3. Nivolumab and Regorafenib

Another study attempting to combine an ICI with a TKI was the single-arm phase 2 RENOBATE trial [19]. The 42 patients on this trial received nivolumab in combination with regorafenib. The primary endpoint was ORR, which was 31%. Additional stable disease was observed in 50% of subjects. The median PFS was 7.38 months, and median OS was not reached, with a 1-year OS rate of 80.5%. Grade 3–4 adverse events were reported in 24% of patients. The side-effect profile was in keeping with those previously described for regorafenib and nivolumab. This trial included a variety of correlative scientific analyses aiming at studying the behavior of different immune cells as well as gene expression patterns.

## 5. Second-Line ICI Therapy in Advanced HCC

While the use of ICIs in the second-line systemic setting initially established its efficacy in HCC, this is less relevant currently, since a variety of ICI-containing approaches are utilized in the frontline systemic setting. Nevertheless, we will discuss the hallmark trials, if for the sake of historic consistency (Table 3).

### 5.1. Nivolumab

The efficacy of nivolumab in the second line was described above [6]. The relevant data are also reflected in Table 1.

### 5.2. Nivolumab and Ipilimumab

One of the cohorts of CheckMate 040 [20,21] investigated the use of nivolumab and ipilimumab in the second-line setting, in patients who failed or were intolerant of sorafenib. This was a randomized, multicenter, open-label phase 1/2 study. The 148 patients were randomized into three arms that differed in doses and frequencies of the investigational drugs. Virtually all patients had Childs–Pugh Class A cirrhosis and 64% were Asian. While vascular invasion was noted in 27–39% of patients (consistent with other cited trials), extrahepatic spread was more common and present in about 80% of subjects. Only a minority of patients, 18–26%, were uninfected with hepatitis B or C. Virtually all patients were pretreated with sorafenib, and most experienced disease progression (about 78–88%) while the remainder were intolerant. After 62.6 months of follow-up, arm A (nivolumab 1 mg/kg and ipilimumab 3 mg/kg every 3 weeks followed by nivolumab 240 mg every 2 week maintenance) had numerically superior outcomes, with ORR of 34%, DOR of 51.2 mos, median OS of 22.2 mos, and a 60 mos OS rate of 29%. In all three arms, survival in responders far exceeded that of non-responders. Time to response was similar in all three arms, about 2–3 months. Serious TRAEs were seen in 22% of patients, and 20% had to discontinue the investigational regimen for this reason.

### 5.3. Pembrolizumab

Keynote-224 was a phase 2, non-randomized, open-label trial that investigated the efficacy of pembrolizumab in sorafenib-pretreated patients [22,23]. It recruited 104 subjects. Unlike the CheckMate 040 trial, most patients (80%) were White. Similar to CheckMate 040, 80% of patients experienced disease progression on sorafenib. In total, 64% of patients had extrahepatic disease, and 17% had macrovascular invasion. After 45 months of follow-up, ORR was 18% and DOR was 21 mos. Median time to response was about 2 months. Median OS was 13.2 mos. The overall survival rate at 36 months was approximately 20%. Grade 3–4 TRAEs were observed in 25% of patients.

KEYNOTE-240 was a randomized, double-blind, phase 3 international trial comparing the efficacy of pembrolizumab with best supportive care alone (BSC) in a second-line systemic setting [24]. It enrolled 413 patients (278 in the pembrolizumab arm) from a variety of geographic areas. Primary endpoints were OS and PFS. Median follow-up was 13.8 mos. Median OS was 13.9 mos in the pembrolizumab arm vs. 10.6 mos in the BSC arm. Median PFS was similar, 3 vs. 2.8 mos. Both of these were numerically higher in the pembrolizumab arm but did not reach statistical significance. ORR was 18.3% vs. 3.3% in the pembrolizumab vs. placebo arms, and median time to response with pembro was 2.7 mos. Any grade 3–4 toxicities were reported in 52% of pembrolizumab-treated and 46% of placebo-treated patients. Adverse events led to treatment discontinuation in 17.2% and 9% of patients, respectively. The high rate of grade 3–4 adverse events in the placebo arm attests to the fact that these patients were medically fragile. The lack of statistical significance of the survival differences in this trial could possibly be explained by the statistical design and boundaries of significance given the dual primary endpoint. Interestingly, the similarly designed KEYNOTE-394 trial conducted in an entirely Asian population did show significant superiority of pembrolizumab in this setting.

### 5.4. Nivolumab and Cabozantinib ± Ipilimumab

This combination was evaluated in Cohort 6 of the CheckMate 040 trial [25]. This cohort enrolled 71 patients, 36 in the doublet and 35 in the triplet arm. Vascular invasion was seen in 39 and 43%, respectively, while extrahepatic spread was seen in 47 vs. 66%. Viral hepatitis-uninfected patients comprised 58% and 40%, respectively. Median follow-up was 32 months. The ORR was 17% and 29%, respectively. While the numbers of subjects are low in this trial, it seems that more nivo-ipi-treated patients responded in a similar setting, 34%. The median OS lasted 20.2 and 22.1 mos, respectively. The duration of response by blinded independent central review was 21.6 mos vs. not reached for the two arms. Given the small number of patients in this early-stage trial, no clinically relevant conclusions can be drawn.

## 6. ICI Therapy for Advanced HCC in Childs–Pugh Class B Patients

Virtually all the patients recruited in the aforementioned trials had well-preserved liver function and were classified as Childs–Pugh Class A, 5 or 6 points. However, treatment of individuals with HCC and underlying more advanced cirrhosis is a very relevant practical concern.

The use of nivolumab in such patients was explored in CheckMate Cohort 5 [26]. This trial enrolled 25 sorafenib-naïve and 24 sorafenib-experienced patients, with 41% having extrahepatic disease and 29% having vascular invasion. Forty-one percent of patients were uninfected by viral hepatitis. The median follow-up was 16.3 mos. The ORR was 12%, DCR was 55%, median time to response was 2.7 mos and median DOR was 9.9 mos. Median OS was 7.6 mos. Significantly, more than half of the responders had an improvement in their liver function to Childs–Pugh Class A. Grade 3–4 TRAEs were reported in 24% of patients, which appeared to be similar to Class A patients treated with nivolumab in other arms of the CheckMate 040 trial.

A systematic review and meta-analysis of the use of ICI monotherapy in Childs–Pugh Class B cirrhotic patients [27] compiled data from 22 clinical trials (19 of 22 being retrospective trials) comprising 699 Class B and 2114 Class A patients, including patient-level data for the 7 largest contributing trials. Class B patients had pulled ORR of 14%, DCR of 46% and median OS of 5.49 mos. When compared to the Class A individuals, Class B patients had inferior ORR (odds ratio, 0.59) and DCR (odds ratio, 0.64); both were statistically significant. The OS was inferior, with HR for dying being 2.72. However, there was no increase in TRAEs. Given no increase in side effects, these results favorably compare to meta-analysis-level data for sorafenib in Class B patients, including ORR of 4.2% and OS of 4.6 mos. The OS in responders was not reported. The preserved safety of ICI monotherapy in Class B patients makes this an attractive therapeutic approach.

## 7. ICI Therapy for Advanced HCC: Choice of Regimen

As can be seen from the presented data, checkpoint inhibitors clearly confer survival advantage in the first-line setting, compared to TKIs such as sorafenib and lenvatinib. Consequently, ICIs should be considered in all patients with good liver function, unless clear contraindications are present. A doublet of PD(L)1-targeting agents plus either an anti-CTLA-4 drug (ipilimumab or tremelimumab) or an anti-VEGF antibody bevacizumab awards remarkable response and survival outcomes. Which combination is best? IMBrave150 was the only choice until more recently. It is difficult to draw inter-trial comparisons, but overall survival outcomes are similar in the sorafenib arms in both the IMbrave140 and Himalaya trials, while OS in the atezo-bev arm appears at least numerically superior, 19.2 vs. 16.4 mos, accompanied also by superior ORR. TRAEs were higher with the atezo-bev combination, although treatment discontinuation rates were similar. In patients with significant portal hypertension, avoiding an anti-VEGF drug may be a prudent strategy to avoid the risk of bleeding. As of April 11, 2025, the combination of nivolumab and ipilimumab has been FDA-approved for previously untreated patients with advanced HCC. The data from CheckMate 9DW have not been presented in a peer-reviewed publication yet; the more cautious reader might await such publication before utilizing this regimen in practice. That said, this combination has been available in the second-line setting for a long time and a lot of oncologists are comfortable using it.

While single-agent ICIs are less effective than combination regimens, they are better tolerated and are likely to be preferred in less-fit patients or those at higher impact of potential autoimmune side effects. Combination regimens should not be offered to Childs–Pugh Class B patients.

The approval for ICIs in patients with advanced HCC is PD-L1 status-agnostic. This is backed up by the data described here: although outcomes are numerically better in patients with PD-L1-expressing tumors, therapeutic benefit is clearly seen in those with PD-L1-negative tumors as well. This was noted for both single-agent and combination regimens [6,8,10]. The survival benefit in PD-L1-negative patients appears to be lacking on subgroup analysis of the IMBrave150 trial, in contrast to the other reports. The significance of this difference is unclear. The methodology, such as basing PD-L1 status on CPS or TPS, further complicates interpretation, since the immunologic milieu of the environment and not just the tumor cells plays an important role.

## 8. ICI Therapy in the Adjuvant Setting After Surgical Resection or RFA

The role of ICIs in the adjuvant setting following liver-directed therapies is also being actively investigated, although data is immature and no overall survival outcomes are available. This application does not constitute standard of care and should not be offered outside of the clinical trial setting. The data below will focus on the available preliminary reports of recurrence-free survival.

### 8.1. Atezolizumab and Bevacizumab 

IMbrave050 was a global, open-label phase 3 study that evaluated the effect of 12 months of adjuvant atezo-bev on survival outcomes in patients with high-risk HCC who underwent curative liver resection or ablation for HCC vs. active surveillance, with 334 subjects in each arm [28]. The majority of patients on this trial, 80%, were recruited in Asia. Over 60% had hepatitis B infection. The population was at higher risk for recurrence than the previously reported STORM trial comparing sorafenib to placebo in a similar clinical setting [29]. With median follow-up of 17.4 mos, the 12-month recurrence-free survival (RFS) rate was significantly superior in the intervention arm, 78 vs. 65%. This is in contrast to the STORM trial which did not show an improvement in RFS. This came at a price: grade 3–4 adverse events occurred in 41% of individuals in the intervention arm vs. 13% in the control arm. Six patients vs. one individual died due to adverse events. The survival data is immature, and in the absence of such data the use of such adjuvant therapy is premature.

### 8.2. Nivolumab 

NIVOLVE was a smaller, phase 2 trial of adjuvant nivolumab for 1 year after curative resection (n = 33) or RFA (n = 22). The results were presented in an abstract form at GI ASCO 2022 [30]. The primary endpoint was 1-year RFS rate. The 12-month RFS rate in this trial was 78.6%. A variety of correlative science observations were made connecting the risk of recurrence with immunosuppressive tumor environment. Further, controlled trials are needed.

### 8.3. Retrospective, Real-World Data with Various Anti-PD(L)1 Drugs 

A real-world, multicenter retrospective database of 627 patients with HCC, treated at seven different hospitals in China, who underwent curative-intent resection was analyzed. Of these, 109 patients received adjuvant immunotherapy and the rest did not [31]. The patients who received adjuvant therapy started it 4–6 weeks after resection; those with R1 resection were excluded. A variety of anti-PD1 or anti-PDL1 agents were used, and duration of therapy was 1 year. The primary endpoints were RFS and OS from time of surgery. A propensity score matching analysis was conducted, including characteristics of the patients, their liver disease, and their tumors. Over half of the systemically treated patients received tislelizumab. The patients in this study were well matched. Over 75% were infected with hepatitis B; 90% were Childs–Pugh Class A, and postoperative TACE was performed in about 30% of patients. The analysis of propensity-matched datasets showed that postoperative immunotherapy was associated with superior RFS (median RFS, 29.6 mos vs. 19.3 mos, *p* < 0.001) and OS (median OS, 35.1 vs. 27.8 mos, *p* < 0.001). This effect was seen in 2-year RFS and OS rates as well. The incidence of immune-related adverse events was 93%, but none are reported to be more than grade 2. A prospective, randomized trial with non-hepatitis-B-related HCC would be a logical sequel to this report.

## 9. Durvalumab and Bevacizumab in Combination with TACE

The benefit of combining systemic therapy with TACE was evaluated in Emerald-1 [32], a global phase 3 randomized, double-blind, placebo-controlled trial of durvalumab (D+TACE, n = 207) or durvalumab + bevacizumab (D+B+TACE, n = 204) compared to TACE alone (n = 205). Durvalumab was started concomitantly with TACE, and bevacizumab was added afterwards. PFS in the D+B+TACE arm was the primary endpoint. PFS in the D+TACE arm, OS, ORR and TTP were secondary endpoints. The primary endpoint was met, with PFS of 15 mos in the D+B+TACE arm vs. 8.2 mos in the TACE arm. The PFS in the D+TACE arm was not significantly different from the control 10 vs. 8.2 mos). OS data is not mature yet. This again comes at an expense of increased toxicity. Grade 3–4TRAEs were similar in the D-TACE and TACE arms, around 15%, but doubled in the D+B+TACE arm at 32%. The same tendency was true for TRAE-related discontinuations. The long-term PFS and OS results will be instrumental in determining the utility of this approach.

## 10. Emerging Therapies

Novel combinations including ICIs are under investigation in patients with HCC. Of particular interest, an anti-TIGIT antibody, tiragolumab, combined with atezolizumab and bevacizumab improved PFS and ORR when compared to atezo-bev alone in the MORPHEUS-liver phase 1B/2 trial [33]. Encouragingly, the combination appeared no more toxic than the well-established control. A randomized, phase 3 trial, IMbrave152, is underway. Such combinations hold promise of further improving outcomes in this ubiquitous and lethal cancer. Future efforts should focus on HCC patients with MASLD-induced cirrhosis, since this etiology of liver disease is of increasing prevalence and relevance. The emerging role of T cells in the pathogenesis of HCC is being discovered [34]. T-cells specific to cancer-testis antigens (CTAs) and tumor-associated antigens (TAAs) appear to be highly expressed and specific for patients with HCC, unlike those with cirrhosis or viral hepatitis. Such TAA-specific T-cells form the basis for chimeric antigen receptor T (CAR-T)-cell therapy in HCC (reviewed in [35]). This approach may be effective in overcoming checkpoint inhibitor resistance in HCC. The role of NK cells in the immune milieu of the liver is also being actively investigated. NK cells constitute a sizable proportion of the hepatic lymphocytes and interact with a variety of other immune cells (reviewed in [36]). CAR-NK cells can be engineered to recognize TAAs and attack cells bearing them [37].

## 11. Conclusions

ICIs have impacted the care of patients with HCC in a variety of settings, improving outcomes in this prevalent cancer. Various combinations are available, with robust data supporting their use. Significant experience has been accumulated, making such therapies acceptably safe and beneficial.

The interpretation of the clinical trial data is complicated by the subsequent therapies patients received, as well as the nature thereof. The interpretation of overall survival data is also affected by the statistical design. For instance, the responders, or patients with stable disease, appear to have better outcomes than non-responders to ICI therapies, and this translates to the overall survival. The reporting of the whole intention-to-treat population lumps responders with non-responders, thus downplaying some nuanced findings such as the robust survival outcomes in responders. This makes subgroup analyses informative, but these are hampered by low numbers of subjects in various subgroups. Finally, the statistical proof of superiority differs from non-inferiority. It would have been helpful to have non-inferiority in some of these trials, especially the single-agent ICI comparisons with sorafenib, to evaluate these drugs vs. the historic standard, sorafenib.

The choice of therapeutic regimen should be tailored to each patient’s unique comorbidities and resultant risks of complications. Specifically, concern for bleeding may limit the utility of VEGF-active agents. The patients with a clinical concern for immune side effects may be safer if dual checkpoint blockade is not offered. At this time, combining immunotherapy with TKIs has not improved outcomes and appears more toxic. Such regimens should not be offered in HCC at this time. Combining anti-PD(L)1 antibodies with novel agents engaging different immune and non-immune mechanisms, as well as exploring different settings and stages within the realm of HCC, promises to further improve patient outcomes. Such new therapies will likely further change the landscape of systemic therapies of HCC in the future.

## Figures and Tables

**Table 1:** Comparison of phase 3 RCTs of single-agent ICI with sorafenib in frontline setting.

Trial	ICI	Patient Allocation, ICI/Sorafenib	Macrovascular Invasion	Extrahepatic Spread	Viral Hepatitis Etiology	Median Follow-Up	OS, mos	ORR, %	DOR, mos	TRAE Grade 3–4, %	TRAE-Related Discontinuation, %
CheckMate 459	nivolumab	371/372	~33%	~60%	55%	~15 mos	16.4 vs. 14.7	15 vs. 7	23.3 vs. 23.4	Serious TRAE 12 vs. 11%	7 vs. 12
Himalaya	durvalumab	389/389	~25%	~54%	~57%	~49 mos	16.6 vs. 13.8	17 vs. 5.1	16.8 vs. 18.4 *	12.9 vs. 36.9	4.1 vs. 11
RATIONALE-301	tislelizumab	342/332	~15%	~60%	76%	33 mos	15.9 vs. 14.1	14.3 vs. 5.4	36.1 vs. 11	22.2 vs. 53.4	6.2 vs. 10.2

* In the 4-year update, 20.3% vs. 6.2% of participants remained in follow-up and 14.4% vs. 2.6% continued to receive study treatment. The majority (57%) of long-term survivors (36 mos or over) in the durvalumab arm were not on therapy at time of this follow-up, while the majority (56%) of patients in the sorafenib arm moved on to another line of therapy. RCT, randomized controlled trial; ICI, immune checkpoint inhibitor; OS, overall survival; ORR, objective response rate; DOR, duration of response; TRAE, treatment-related adverse event.

**Table 2:** Comparison of phase 3 RCTs of combination therapy including ICI vs. sorafenib in frontline setting.

Trial	Intervention Arm	Patient Allocation, ICI/Control	Macrovascular Invasion	Extrahepatic Spread	Viral Hepatitis Etiology	Median Follow-Up	OS, mos	ORR, %	DOR, mos	All Grade 3–4, %	TRAE-Related Discontinuation, %
IMbrave150	atezo+bev vs. sorafenib	336/165	~40%	~60%	~70%	~20 mos	19.2 vs. 13.4 mos	27.3 vs. 11.9 mos	NA	56.5 vs. 55.1%	15.5 vs. 10.3%
Himalaya	treme+durva vs. sorafenib	393/389	~25%	~53%	~60%	~49 mos	16.4 vs. 13.8 mos	20.1 vs. 5.1	22.3 vs. 18.4	TR SAE 17.5 vs. 9.6	13.7 vs. 16.8 %
CheckMate 9DW	nivo+ipi vs. sorafenib or lenvatinib	335/333	NR	NR	NR	~35 mos	23.7 vs. 20.6 mos	36 vs. 13%	30.4 vs. 12.9 mos	41 vs. 42%	18 vs. 10%

RCT, randomized controlled trial; ICI, immune checkpoint inhibitor; NR, not reported; OS, overall survival; ORR, objective response rate; DOR, duration of response; TRAE, treatment-related adverse event; NA, not applicable; TR SAE, treatment-related serious adverse event.

**Table 3:** Comparison of second-line efficacy in sorafenib-pretreated patients.

Trial	Intervention Arm	Pts, n	MVI	Extrahepatic Spread	Viral Hepatitis Etiology	Median Follow-Up	OS, mos	ORR, %	DOR, mos	TRAE Grade 3–4, %	TRAE-Related Discontinuation, %
CheckMate 040 nivo	nivolumab	145	40%	71%	70%	NR	13.2+ mos	21%	9.9 mos	Serious, 4%	7%
KEYNOTE-224	pembrolizumab	104	17%	64%	Hep B21%, Hep C25%	45 mos	13.2	18%	21 mos	25%	4.80%
KEYNOTE-240	pembrolizumab	278	13%	70%	41%	13.8 mos	13.9 mos	18%	13.8 mos	any grade 3–4, 52%	17.20%
	BSC	135	12%	69%	37%	10.6 mos	10.6 mos	4.40%	not reached	any grade 3–4, 46%%	9%
CheckMate 040, *arm A	nivo-ipi	148	35%	82%	~70%	60 mos	* 22.2 mos	* 34%	*51.2 mos	* 55%, * serious 20%	* 22%

BSC, best supportive care; MVI, macrovascular invasion; NR, not reported; OS, overall survival; ORR, objective response rate; DOR, duration of response; TRAE, treatment-related adverse event.
